# Supplementary figures and images for: Perception of a conserved family of plant signalling peptides by the receptor kinase HSL3
Source: eLife. 2022 May 26;11:e74687. doi: 10.7554/eLife.74687 (PMC9191895; doi:10.7554/eLife.74687)

Figure 1 e

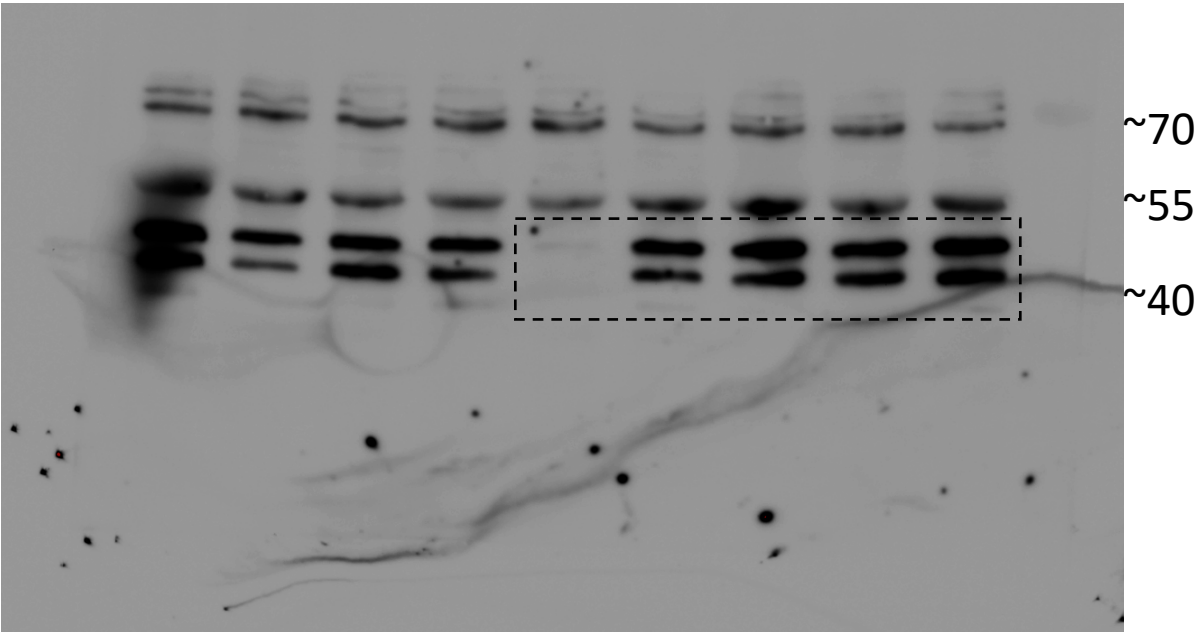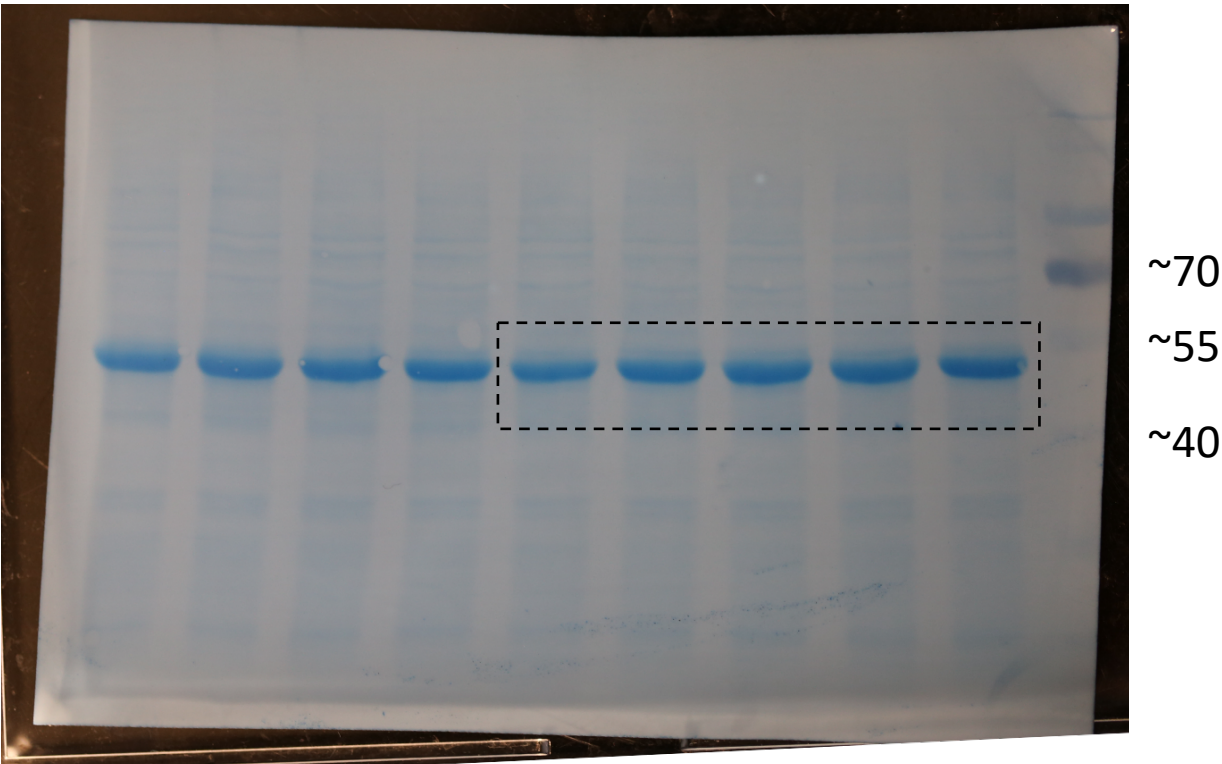

Supplement: Figure 1—source data 2. [file elife-74687-fig1-data2.pdf]

Figure1-figure supplement 2

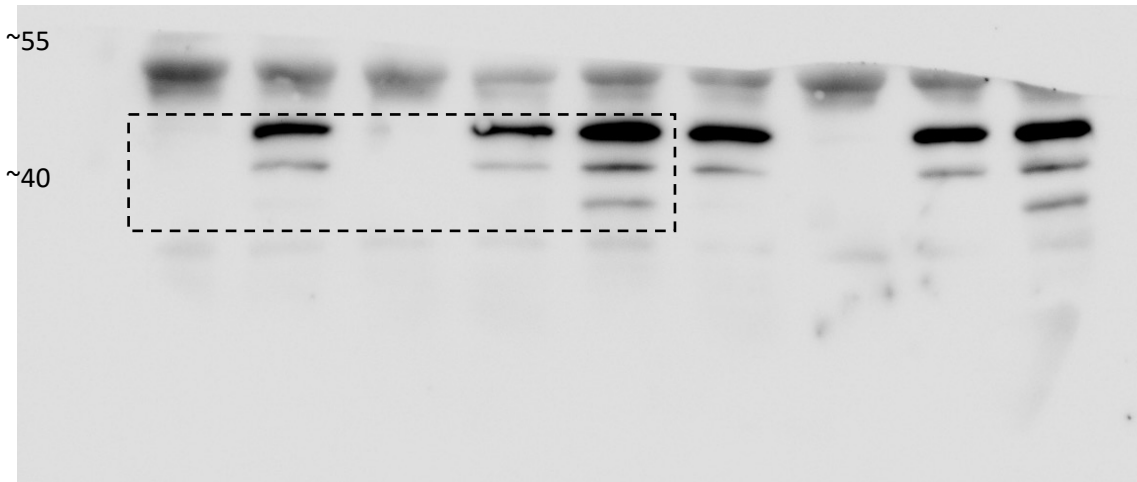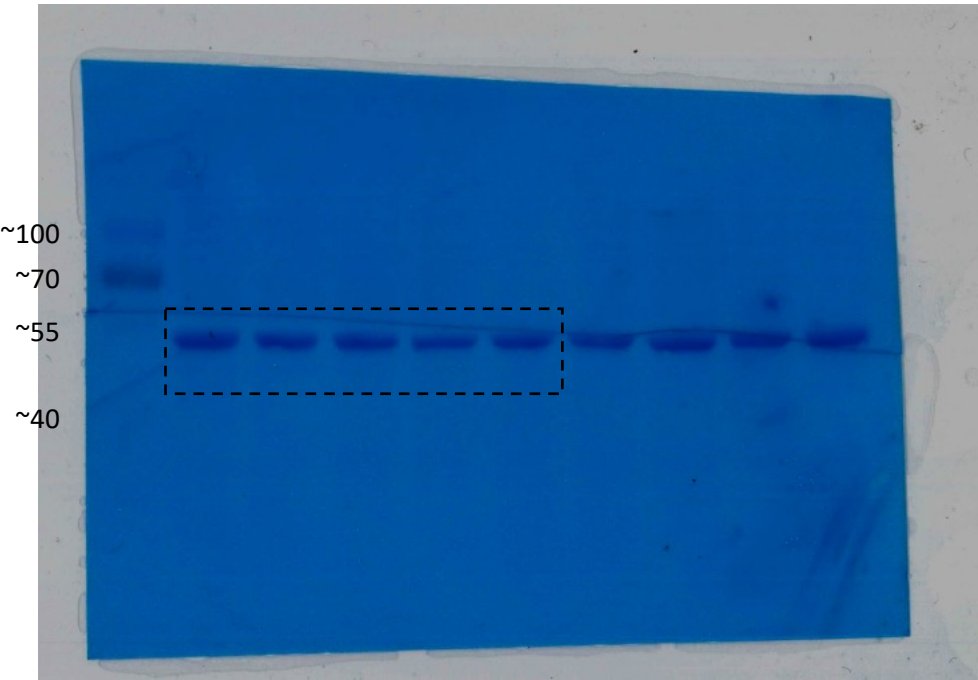

Supplement: Figure 1—figure supplement 2—source data 2. [file elife-74687-fig1-figsupp2-data2.pdf]

Figure 2c

IP-GFP

-HSL3-GFP

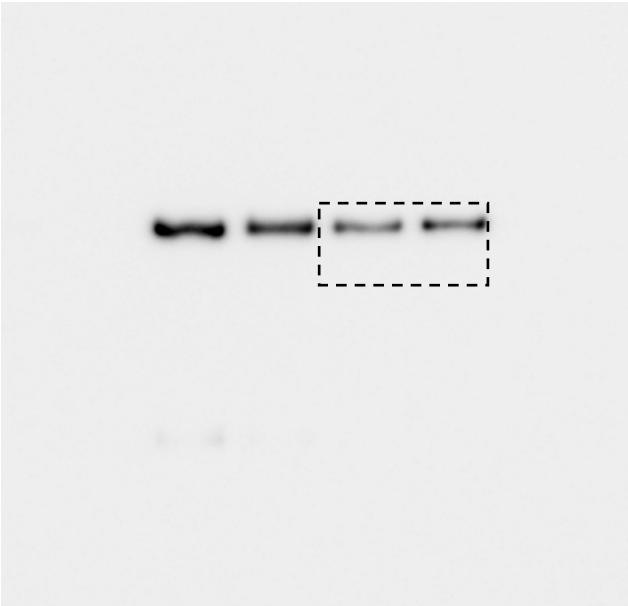

$\alpha$  GFP

-BAK1

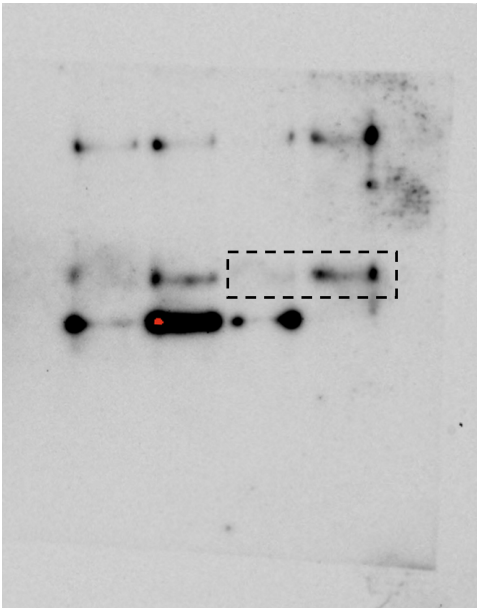

$\alpha$  BAK1

Input

-HSL3-GFP

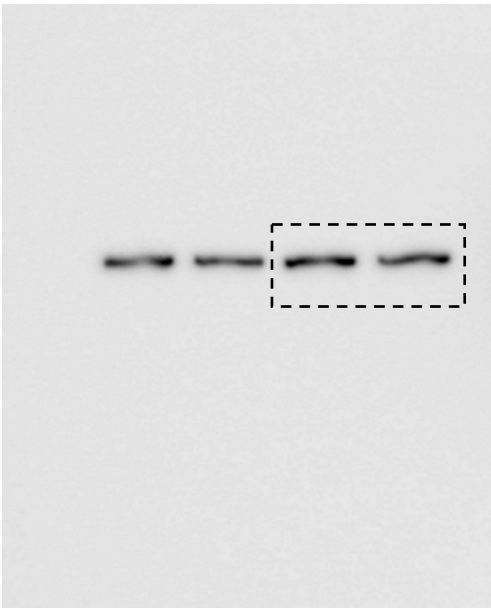

$\alpha$  GFP

-BAK1

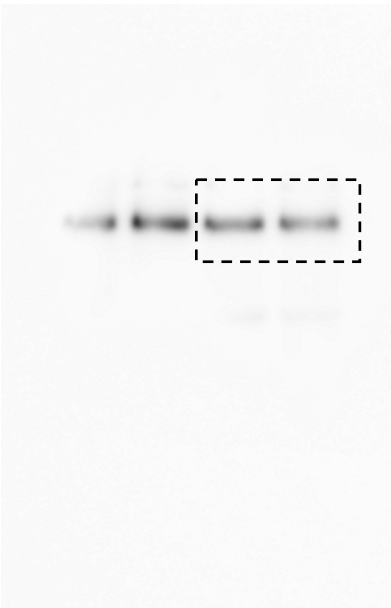

$\alpha$  BAK1

Supplement: Figure 2—source data 2. [file elife-74687-fig2-data2.pdf]

Figure 3 a

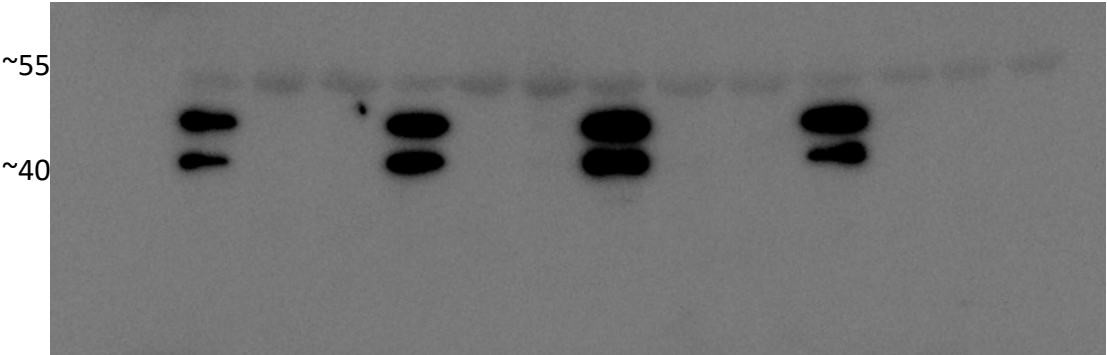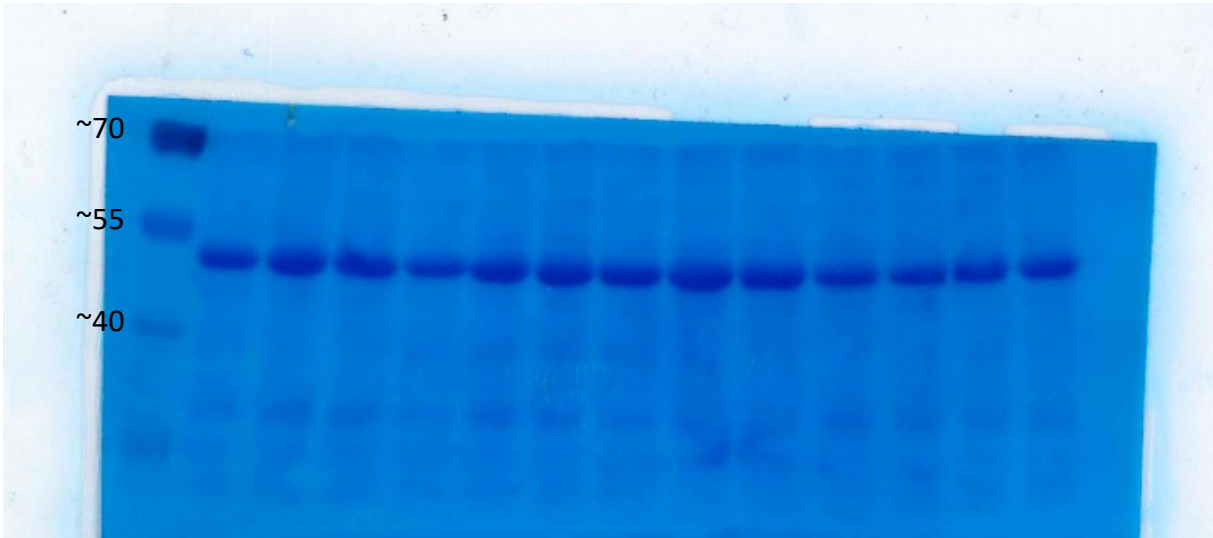

Supplement: Figure 3—source data 2. [file elife-74687-fig3-data2.pdf]

Figure 3-figure supplement 1

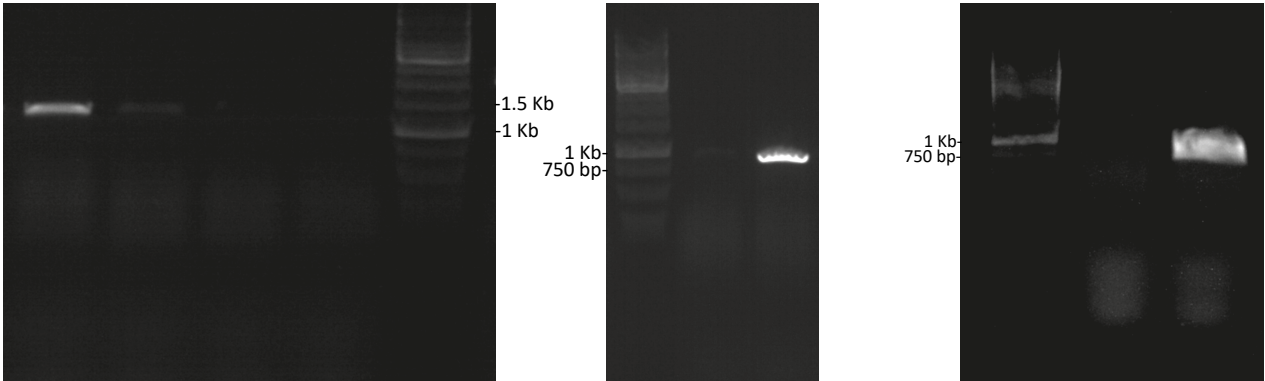

Supplement: Figure 3—figure supplement 1—source data 1. [file elife-74687-fig3-figsupp1-data1.pdf]

Figure3-figure supplement 4

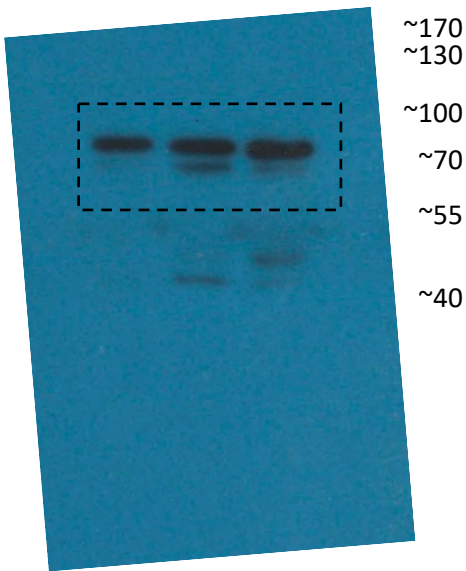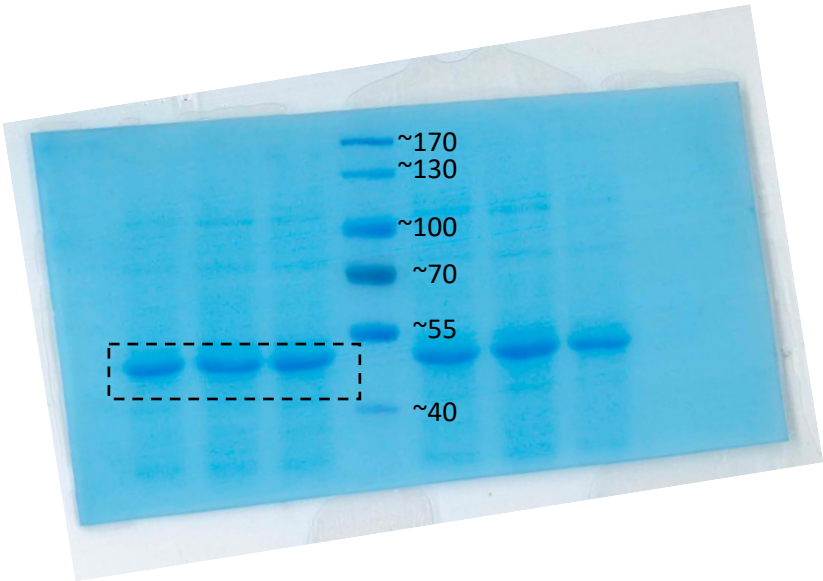

Supplement: Figure 3—figure supplement 4—source data 1. [file elife-74687-fig3-figsupp4-data1.pdf]
