# Supplementary material for: Perception of a conserved family of plant signalling peptides by the receptor kinase HSL3
Source: eLife. 2022 May 26;11:e74687. doi: 10.7554/eLife.74687 (PMC9191895; doi:10.7554/eLife.74687)
Supplement: Supplementary file 6. [file elife-74687-supp6.docx]

| **Peptide name** | **Sequence** | **Length** | **Supplier** |
| --- | --- | --- | --- |
| CTNIP1 (AT1G06135) | AMRPFPTAADEIRFVFQALQRGPVSGSGPNGCTNIPRGTPRCHG | 44 | EZBiolab |
| CTNIP2 (AT1G06137) | AARPLQADSEIRFVFQLLQRGQVIGSGPNGCTNIPGGSGTCRP | 43 | EZBiolab |
| CTNIP3 (AT2G31335) | ATRMLRITFDSDIRFVFESLQKGTVPGSGPNRCSHIPKGSGSCHG | 45 | EZBiolab |
| CTNIP4 (AT2G31345) | AMRPFPDPVDEIRLLFQALQRGPVRGSGRNGCTNIPRGSGRCHN | 44 | EZBiolab |
| SCOOP12 | PVRSSQSSQAGGR | 13 | EZBiolab |
| CTNIP4^48-70^ | GPVRGSGRNGCTNIPRGSGRCHN | 23 | Genscript |
| CTNIP4^C58S^ | GPVRGSGRNGSTNIPRGSGRCHN | 23 | EZBiolab |
| CTNIP4^C68S^ | GPVRGSGRNGCTNIPRGSGRSHN | 23 | EZBiolab |
| CTNIP4^C58S/C68S^ | GPVRGSGRNGSTNIPRGSGRSHN | 23 | EZBiolab |
| CTNIP5 (AT3G23123) | GEVTPSNPSSCTHIPGGHGPPCP | 23 | EZBiolab |
| *Mt*CTNIP (Medtr1g044470) | KGPVAPSGPSGCTFIPGSGGTHCPIEERN | 29 | EZBiolab |
